# 

**Published:** 1899-04

**Authors:** 


### CONTENTS.

COMMUNICATIONS.

Dental Nomenclature ........ 151
Dental Legislation ........ 162
The Physiology and Pathology of the Peridentium ........ 164
Falsetto Voice in the Male ........ 180
The Maxillary Sinus—Chronic Empyema Thereof; Its Treatment... 186

PROCEEDINGS.

Southern Branch, National Dental Association, Second Annual Meeting, New Orleans, La., February 9, 10, 11 and 13, 1899...... 169
"Orthodontia and Oral Surgery" ........ 173
"Conservative Antrum Treatment" ........ 174
"A Simplified Fracture Splint" ........ 176
"A Case in Oral Surgery" ........ 177

SELECTIONS.

Appropriation for the John Hopkins University........ 161
The Prevention of Disease........ 190
Orthoform for Toothache........ 193
Are Athletes Healthy?........ 194
Hygienic Value of the Sun's Rays........ 195
Unnatural Death........ 196
Ten Prize Hygienic Rules........ 197
Jaborandi an Exceedingly Valuable Remedy ........ 197
Recovery of a Lost Nose ........ 198
Medical College Consolidation ........ 200

NOTICES.

The Northern Ohio Dental Association........ 198

EDITORIAL.

Bibliographical ........ 199

